# Comparison of the MGISEQ-2000 and Illumina HiSeq 4000 sequencing platforms for RNA sequencing

**DOI:** 10.5808/GI.2019.17.3.e32

**Published:** 2019-09-27

**Authors:** Sol A Jeon, Jong Lyul Park, Jong-Hwan Kim, Jeong Hwan Kim, Yong Sung Kim, Jin Cheon Kim, Seon-Young Kim

**Affiliations:** 1 Personalized Genomic Medicine Research Center, Korea Research Institute of Bioscience and Biotechnology, Daejeon 34141, Korea; 2 Department of Bioscience, University of Science and Technology, Daejeon 34113, Korea; 3 Genome Editing Research Center, Korea Research Institute of Bioscience and Biotechnology, Daejeon 34141, Korea; 4 Department of Surgery, University of Ulsan College of Medicine, Seoul, Korea; 5 Department of Cancer Research, Institute of Innovative Cancer Research and Asan Institute for Life Sciences, Asan Medical Center, Seoul 05505, Korea

**Keywords:** benchmarking, HiSeq 4000, MGISEQ-2000

## Abstract

Currently, Illumina sequencers are the globally leading sequencing platform in the next-generation sequencing market. Recently, MGI Tech launched a series of new sequencers, including the MGISEQ-2000, which promise to deliver high-quality sequencing data faster and at lower prices than Illumina’s sequencers. In this study, we compared the performance of two major sequencers (MGISEQ-2000 and HiSeq 4000) to test whether the MGISEQ-2000 sequencer delivers high-quality sequence data as suggested. We performed RNA sequencing of four human colon cancer samples with the two platforms, and compared the sequencing quality and expression values. The data produced from the MGISEQ-2000 and HiSeq 4000 showed high concordance, with Pearson correlation coefficients ranging from 0.98 to 0.99. Various quality control (QC) analyses showed that the MGISEQ-2000 data fulfilled the required QC measures. Our study suggests that the performance of the MGISEQ-2000 is comparable to that of the HiSeq 4000 and that the MGISEQ-2000 can be a useful platform for sequencing.

## Introduction

Next-generation sequencing (NGS) technology has had a major impact on the field of genomics since its first release in 2005 [1]. Since then, many different NGS platforms have been developed, adopting different strategies and chemical techniques [1]. However, NGS machines based on Illumina’s sequencing by synthesis method have dominated the sequencing market owing to their high accuracy and high throughput. The NovaSeq 6000, the latest instrument of Illumina’s series, now generates 6 TB of sequence data in a single run with a running cost of 12-18 USD/GB.

Recently, MGI Tech, a subsidiary of the Beijing Genomics Institute (BGI) Group, launched a series of new NGS machines (the BGI-200, BGI-500, MGISEQ-2000, and MGISEQ-T7) based on DNA nanoball technology; these devices promise to deliver high-quality sequencing data faster at lower prices. For example, the MGISEQ-2000 currently generates 1.44 TB of sequence data in a single run with a running cost of 10 USD/GB. Several recent studies have compared the performance of BGI sequencers with Illumina’s sequencers and showed that the BGI sequencers produced high-quality sequence data at lower or similar prices in studies of whole-exome [2,3], whole-genome [4][1], transcriptome [5,6], single-cell transcriptome [2,7,8], metagenome [9], and small RNA sequencing [10].

In this study, we compared the performance of MGISEQ-2000 with that of Illumina’s HiSeq 4000 by sequencing the same RNAs from four human colorectal cancer patients’ tissue samples. We found that the MGISEQ-2000 produced high-quality sequence data comparable to the data obtained by the HiSeq 4000, at half the price. We suggest that the MGISEQ-2000 is a promising sequencing platform for whole-transcriptomics studies with high performance and low cost.

## Methods

### RNA extraction, library construction, and sequencing

Total RNA was isolated from four human colon tissue samples using an RNeasy Blood and Tissue kit (Qiagen, Carlsbad, CA, USA). To construct the sequencing library for HiSeq 4000, we followed the TruSeq Stranded mRNA Sample Preparation Guide, Part #15031047 Rev. E. Approximately 2 μg of total RNA was used for library construction with the Illumina TruSeq Stranded mRNA Library Prep Kit (San Diego, CA, USA). Next, paired-end sequencing was performed using the Illumina HiSeq4000 sequencing instrument, according to the manufacturer’s instructions, yielding 101-bp paired-end reads. To construct the library for the MGISEQ-2000, approximately 1 μg of total RNA was used for library construction using the MGIEasy RNA Directional Library Prep Kit (MGI). Next, paired-end sequencing was performed using the MGISEQ-2000 sequencing instrument, according to the manufacturer’s instructions, yielding 100-bp paired-end reads. The RNA-seq data of HiSeq 4000 were generated in 2013, while the MGISEQ-2000 data were generated in 2019. Thus, although we used RNA from the same samples, the sequencing was not performed at the same time.

### Sequencing quality check, mapping, and data analysis

We used FastQC v0.11.5 to check the quality of the sequencing results. The simple Python script q30 (https://github.com/dayedepps/q30) was used to calculate the exact percentages of Q20/Q30. We used STAR_2.5.4b, an ultrafast universal RNA-seq aligner, to align the RNA-seq data onto the hg19 reference genome [11]. We ran the mapping job with the quantMode set as the GeneCounts option. Using the R statistical language, we normalized the read count data and converted its scale into the base 2 logarithm of counts per million (cpm). A scatter plot was drawn using ggscatter, one of the functions of the R package ggpubr. Correlation graphs were drawn using Microsoft Excel 2013. The data used in drawing scatter plot and correlation graphs were normalized and converted into the base 2 logarithm of cpm, as mentioned above. To obtain Venn diagrams of the upregulated differentially expressed genes (DEGs) and the downregulated DEGs, we used jvenn, an interactive Venn diagram viewer (http://jvenn.toulouse.inra.fr/app/index.html) [12].

## Results and Discussion

### Comparison of sequencing and mapping data quality

We sequenced four human colon tumor tissue samples with Illumina’s HiSeq 4000 and the MGISEQ-2000, and checked the quality of the sequences by running the FastQC program. Overall, the sequence quality of the two platforms was similar. In terms of the Phred score, the MGISEQ-2000 showed a higher percentage for over-Q20 bases, but a lower percentage for over-Q30 bases than the Illumina HiSeq 4000 (Table 1). For over-Q20 bases, the HiSeq 4000 showed an average of 97.84% and the MGISEQ-2000 showed an average of 98.20%. For over-Q30 bases, the HiSeq 4000 showed an average of 94.63% and the MGISEQ-2000 showed an average of 92.60%. For uniquely mapped reads, the MGISEQ-2000 produced better mapping results than the HiSeq 4000 in all four samples (Table 1). On average, the sequencing reads from the MGISEQ-2000 mapped 2.3% more data than the HiSeq 4000.

### Concordance between the MGISEQ-2000 and HiSeq 4000

**Fig. 1.**
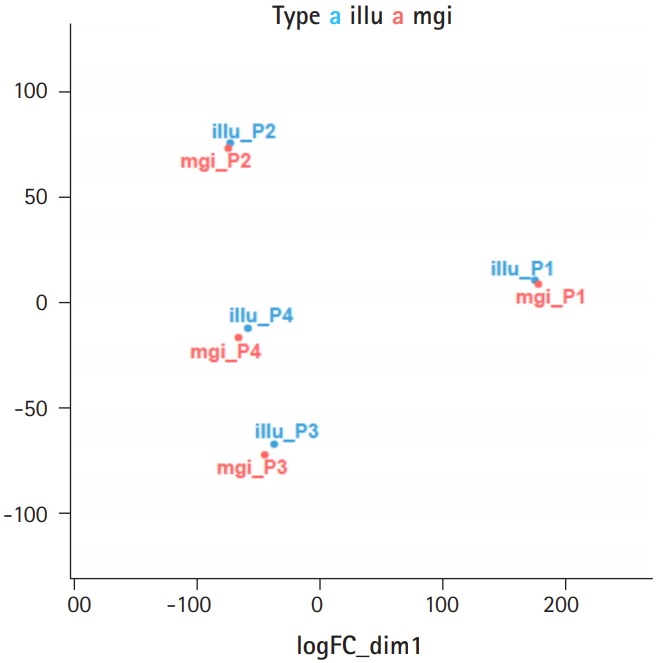
High concordance of RNA-seq data produced using the Illumina and MGI platforms as shown by a principal component analysis plot. RNA from the four samples was sequenced using the HiSeq 4000 (blue dots) and MGISEQ-2000 (red dots) sequencers.

**Fig. 2.**
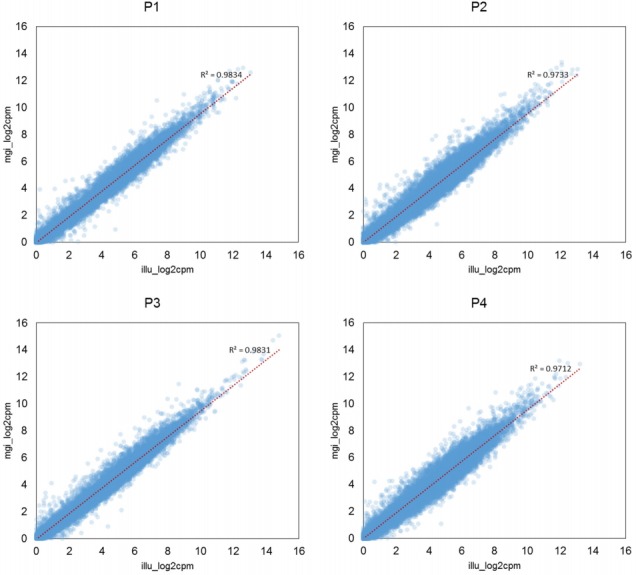
Scatter plots of gene expression values of the four pairs of samples produced using the HiSeq 4000 and MGISEQ-2000 sequencers. Gene expression values are represented as the base 2 logarithm of counts per million (cpm). The Pearson correlation coefficients of the four samples were between 0.98 and 0.99.

We checked the concordance of the RNA-seq data produced by the two platforms using two methods: principal component analysis (PCA) of the eight samples, and pairwise correlation analysis (Supplementary Fig. 1). When we performed PCA of the eight samples, we found that the four pairs of samples were located close to each other, showing that no significant biases existed between the two sequencing platforms (Fig. 1). Then, we calculated the Pearson correlation coefficient of the four pairs and found that all four pairs of samples showed high correlation coefficients, ranging from 0.98 to 0.99 (Fig. 2). Thus, we found that the MGISEQ-2000 and HiSeq 4000 produced highly reproducible data from the same samples without significant platform-specific biases.

### DEGs between the MGISEQ-2000 and HiSeq 4000

**Fig. 3.**
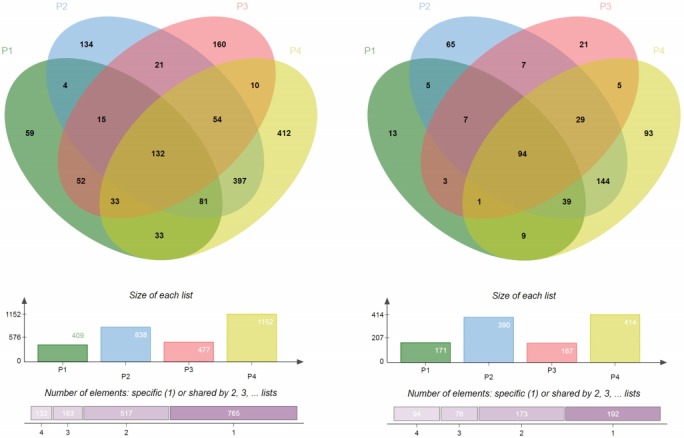
Differentially expressed genes between the two platforms. Genes with larger than two-fold differences were selected from the four pairs of samples. As only one experiment was performed for each platform, no statistical test was applied. The overlap of the differentially expressed genes is shown.

We observed a small number of DEGs (fold change over two) between the MGISEQ-2000 and HiSeq 4000 platforms (Supplementary Tables 1–4), but most of them were random DEGs without systematic bias (Fig. 3). Among the four pairs of samples (P1, P2, P3, and P4), there were 409, 838, 477, and 1152 downregulated DEGs, and 171, 390, 167, and 414 upregulated DEGs, respectively. We further searched for overlapping genes and found that there were 132 downregulated DEGs and 94 upregulated DEGs that were common among the four pairs of samples (P1, P2, P3, and P4). In detail, among the downregulated DEGs in P2, 664 of 839 genes (approximately 80%) (Fig. 3, Supplementary Table 5) were also downregulated in P4. Considering that P4 had many downregulated DEGs compared to other samples, it still showed quite a high percentage of intersection with P2 (about 58%) (Fig. 3, Supplementary Table 5). For upregulated DEGs, we also noticed that P2 and P4 shared a substantial proportion of upregulated DEGs (over 70%) (Fig. 3, Supplementary Table 6), even though they had more upregulated DEGs than the other samples (P1, P3). As we conducted a gene ontology analysis, we found that ribosomal protein-coding genes showed some tendency to be present among the downregulated DEGs (Supplementary Table 7), while genes related to transcription showed a slight tendency to be present among the upregulated DEGs (Supplementary Table 8). However, as mentioned in the Methods section, we did not generate the two sets of RNA-seq data at the same time, leading to the concern that some degradation of the RNA samples may have taken place over the 6-year interval. Another limitation is that we sequenced each sample for each platform once without duplicates, which may have increased the likelihood of errors.

While sequencing costs have declined significantly over the years, the ever-increasing sample size and scale of omics projects necessitate the use of sequencing technology with lower costs. In this regard, sequencing instruments such as the BGI-500, MGISEQ-2000, and MGISEQ-T7 are attractive alternatives to Illumina’s HiSeq and NovaSeq series, as they enable researchers to generate the same amount of data at lower costs. Several recent papers have compared the performance of the BGI-500 with that of Illumina’s HiSeq machines and showed that both machines produced high-quality data in diverse applications such as whole-exome [3], whole-genome [13-15], small RNA [10], and metagenome sequencing [9], as well as plant-tissue transcriptomics [5] and single-cell transcriptomics [7,8]. In this study, we also found that the MGISEQ-2000 and HiSeq 4000 produced highly concordant gene expression data from the four colorectal tumor tissue samples. While the two platforms exhibit similar base sequencing quality, we found that the MGISEQ-2000 produced sequencing data with higher mapping quality than the HiSeq 4000 in all samples (Table 1). A recent study also reported that the MGISEQ-2000 platform performed consistently better than the NextSeq 500 platform in a single-cell transcriptomics study, detecting more cells, genes, and unique molecular identifiers [8]. They also reported that the MGISEQ-2000 produced more single-nucleotide polymorphism calls from sequence data, enabling an additional 14% of cells to be assigned to the correct donor from a multiplexed library [8]. Thus, we conclude that the MGISEQ-2000 is a robust sequencing platform that produces high-quality sequencing data at lower costs and can be used in many NGS applications.

## Figures and Tables

**Table 1.** Summary statistics of sequencing quality

	Total read bases (bp)		Q20 (%)		Q30 (%)		Uniquely mapped reads (%)	
Illumina	MGI	Illumina	MGI	Illumina	MGI	Illumina	MGI
P1	7.45×10^9^	2.44×10^10^	97.9	98.23	94.75	92.65	93.65	95.74
P2	7.36×10^9^	2.46×10^10^	97.87	98.26	94.67	92.85	89.8	91.8
P3	8.71×10^9^	2.40×10^10^	97.72	98.09	94.36	92.25	93.75	96.6
P4	9.35×10^9^	1.99×10^10^	97.88	98.23	94.73	92.65	92.75	94.65

## Supplementary Materials

Supplementary Fig. 1. Schematic diagram of the experimental and analytics pipeline of this study. DEG, differentially expressed gene; cpm, counts per million.

Supplementary Table 1. Differentially expressed genes from sample 1

Supplementary Table 2. Differentially expressed genes from sample 2

Supplementary Table 3. Differentially expressed genes from sample 3

Supplementary Table 4. Differentially expressed genes from sample 4

Supplementary Table 5. Intersections among downregulated differentially expressed genes

Supplementary Table 6. Intersections among upregulated differentially expressed genes

Supplementary Table 7. Gene ontology analysis of downregulated genes

Supplementary Table 8. Gene ontology analysis of upregulated genes
