# Supplementary figures and images for: Comparison of the MGISEQ-2000 and Illumina HiSeq 4000 sequencing platforms for RNA sequencing
Source: Genomics Inform. 2019 Sep 27;17(3):e32. doi: 10.5808/GI.2019.17.3.e32 (PMC6808641; doi:10.5808/GI.2019.17.3.e32)

Supplementary Figure 1

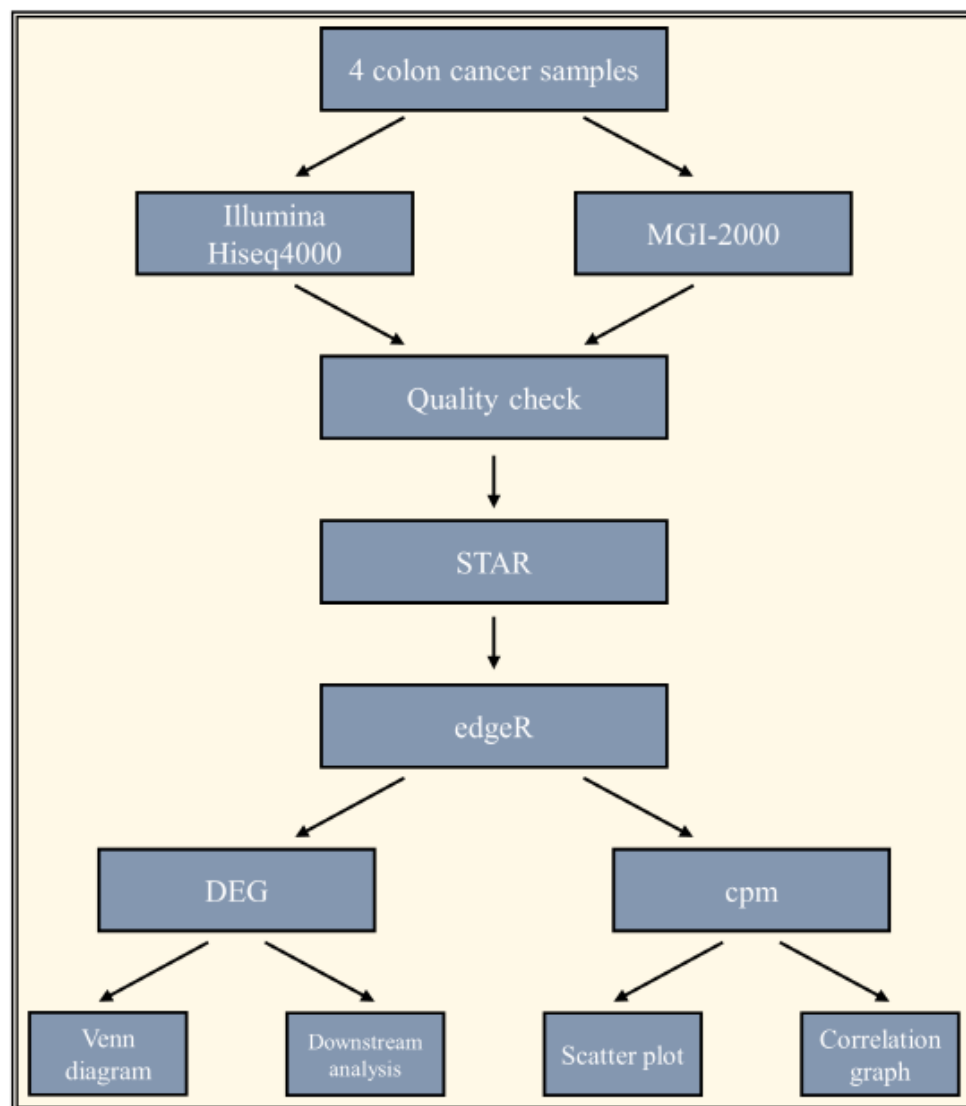

Supplement: Supplementary Fig. 1. — Schematic diagram of the experimental and analytics pipeline of this study. DEG, differentially expressed gene; cpm, counts per million. [file gi-2019-17-3-e32-suppl1.pdf]
