# Supplementary material for: Comparison of the MGISEQ-2000 and Illumina HiSeq 4000 sequencing platforms for RNA sequencing
Source: Genomics Inform. 2019 Sep 27;17(3):e32. doi: 10.5808/GI.2019.17.3.e32 (PMC6808641; doi:10.5808/GI.2019.17.3.e32)
Supplement: Supplementary Table 5. — Intersections among downregulated differentially expressed genes [file gi-2019-17-3-e32-supple6.docx]

Supplementary Table 5. Intersections among down-regulated DEGs

| **Down DEG** | **P1 (409)** | **P2 (838)** | **P3 (477)** | **P4 (1152)** |
| --- | --- | --- | --- | --- |
| P1 ∩ P2 | 56.7% | 27.7% | - | - |
| P1 ∩ P3 | 56.7% | - | 48.6% | - |
| P1 ∩ P4 | 68.2% | - | - | 24.2% |
| P2 ∩ P3 | - | 26.5% | 46.5% | - |
| P2 ∩ P4 | - | 79.2% | - | 57.6% |
| P3 ∩ P4 | - | - | 48.0% | 19.9% |

Supplementary Table 6. Intersections among up-regulated DEGs

| **Up DEG** | **P1 (171)** | **P2 (390)** | **P3 (167)** | **P4 (414)** |
| --- | --- | --- | --- | --- |
| P1 ∩ P2 | 84.8% | 37.2% | - | - |
| P1 ∩ P3 | 61.4% | - | 62.9% | - |
| P1 ∩ P4 | 83.6% | - | - | 34.5% |
| P2 ∩ P3 | - | 35.1% | 82.0% | - |
| P2 ∩ P4 | - | 78.5% | - | 73.9% |
| P3 ∩ P4 | - | - | 77.2% | 31.2% |
